# Supplementary material for: The RNA m5C modification in R-loops as an off switch of Alt-NHEJ
Source: Nat Commun. 2023 Sep 30;14:6114. doi: 10.1038/s41467-023-41790-w (PMC10542358; doi:10.1038/s41467-023-41790-w)
Supplement: Supplementary file 1 — Supplementary Information [file 41467_2023_41790_MOESM1_ESM.pdf]

Supplementary Information for

The RNA m5C Modification in R-loops as an Off Switch of Alt-NHEJ

Haibo Yang[1,2], Emily M. Lachtara[1,3], Xiaojuan Ran[1,3,4], Jessica Hopkins[1,3], Parasvi S. Patel[1,3], Xueping Zhu[5], Yao Xiao[1,2], Laiyee Phoon[6], Boya Gao[1,2], Lee Zou[1,3,4], Michael S. Lawrence[1,3], Li Lan[1,2,6*]

[1]Massachusetts General Hospital Cancer Center, Harvard Medical School, Boston, MA 02129 USA.

[2]Department of Radiation Oncology, Massachusetts General Hospital, Harvard Medical School, Boston, MA 02129 USA.

[3] Department of Pathology, Massachusetts General Hospital, Harvard Medical School, Boston, MA 02114, USA

[4]Department of Pharmacology and Cancer Biology, Duke University School of Medicine, Durham, NC 27708

[5]Center for Immunology and Inflammatory Diseases, Division of Rheumatology, Allergy, and Immunology, Massachusetts General Hospital, Harvard Medical School, Boston, MA 02114

[6]Departments of Molecular Genetics and Microbiology, School of Medicine, Duke University, Durham, NC 27710

[*]To whom correspondence should be addressed: Dr. Li Lan, Departments of Molecular Genetics and Microbiology, School of Medicine, Duke University, 424 CARL Building, Research Drive, Durham, NC 27710 USA. Telephone: 919-613-8626; E-mail: li.lan@duke.edu.

This PDF file includes

Supplementary Figure 1 to Supplementary Figure 8
Supplementary Table 1 to Supplementary Table 3

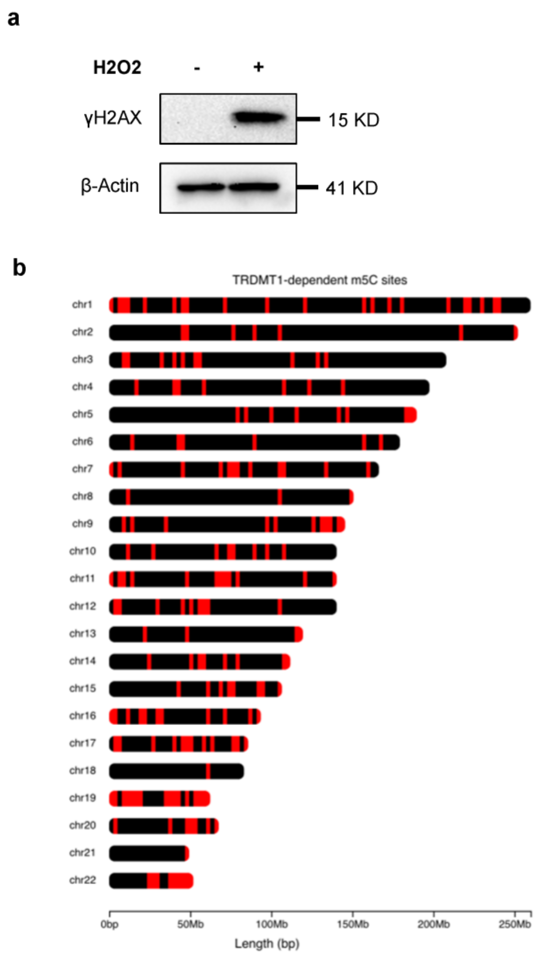

Supplementary Figure 1. (a) U2OS-TRE cells were treated with 1 mM H_2O_2 for 1 h before harvest for WB. The γH2AX was blotted. (b) A chromosome plot detailing the location of the TRDMT1-dependent m5C sites. Source data are provided as a Source Data file.

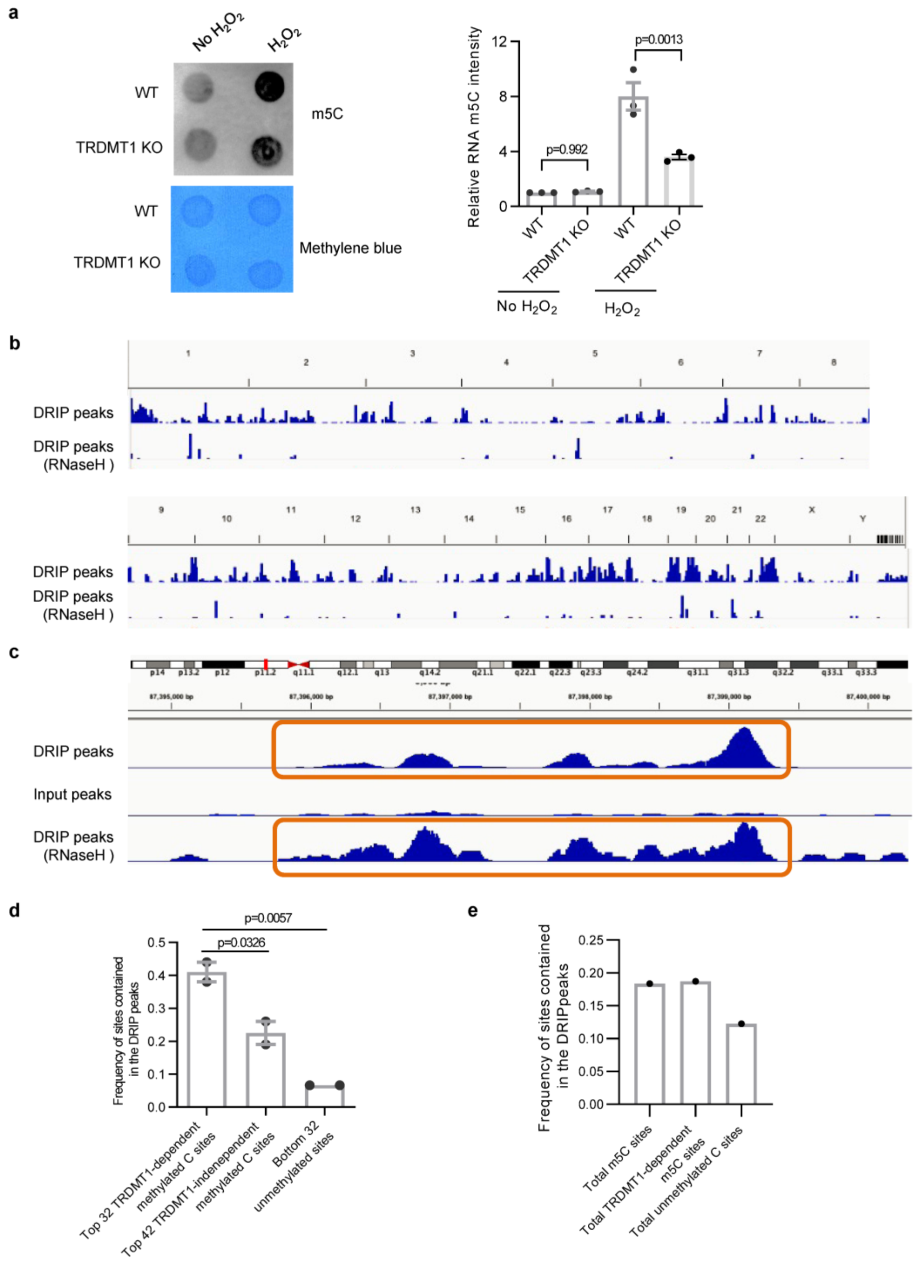

Supplementary Figure 2. (a) The m5C dot blot for the mRNA extracted from U2OS WT and TRDMT1 KO cells with or without H2O2 damage. The cells were treated with 1mM H2O2 for 1 h before extraction for mRNA with Dynabeads™ mRNA DIRECT™ Purification Kit. Methylene blue was used as the loading control. The levels of m5C was quantified (n = 3 independent experiments, Mean ± SEM). (b) An overview of peaks obtained from DRIP-seq. (c) An example of a DRIP peak which was also obtained with RNaseH treatment before sequencing. The RNaseH-resistant peak would be removed from the analysis. (d) The fraction of top 32 TRDMT1-dependent methylated sites or top 42 TRDMT1-independent methylated sites or bottom 32 unmethylated sites contained in the DRIP peaks. (e) The fraction of total m5C sites or total TRDMT1-dependent m5C sites or total unmethylated C sites contained in the DRIP peaks. The Statistical analysis was done with one-way ANOVA. Source data are provided as a Source Data file.

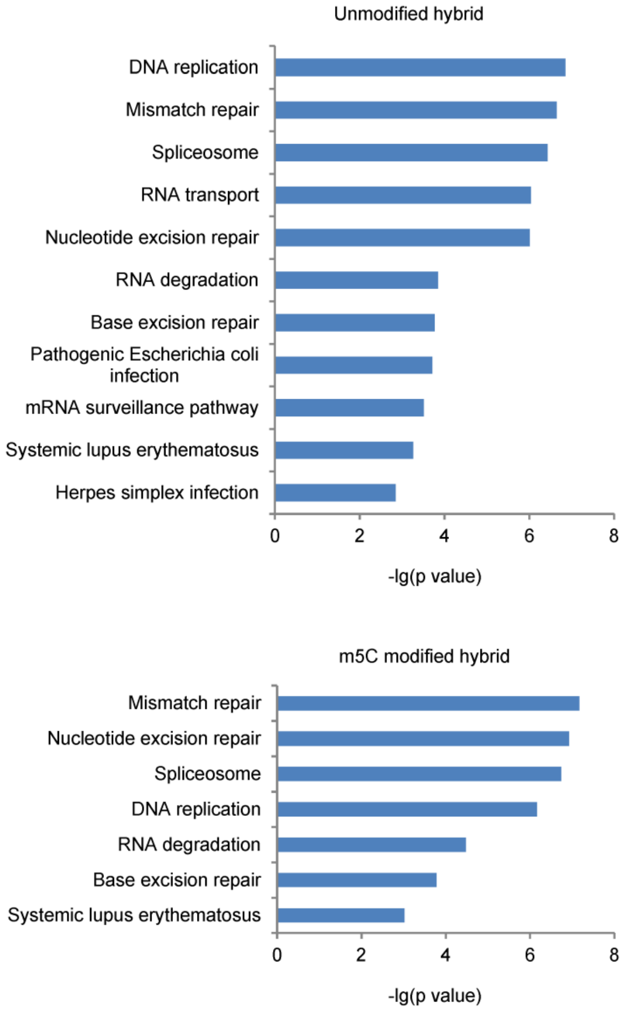

Supplementary Figure 3. KEGG pathway enrichment analysis for the binding partners of unmodified or RNA m5C modified RNA:DNA hybrid.

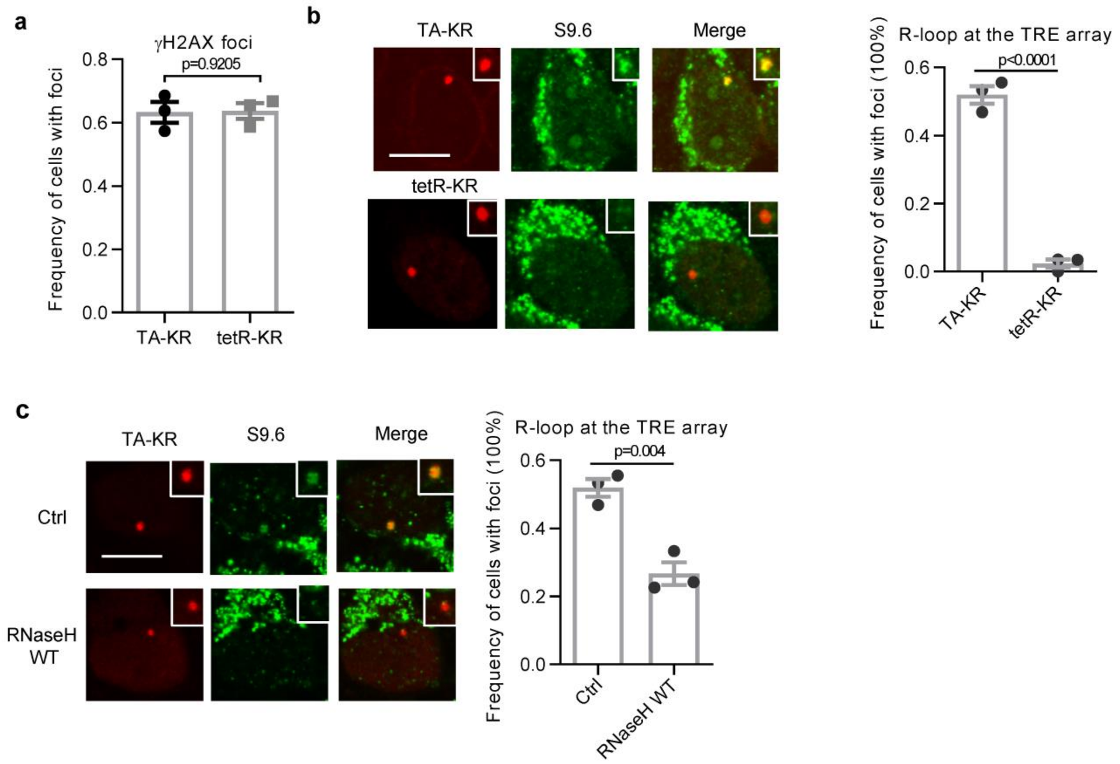

Supplementary Figure 4. m5C RNA modifications suppress the damage response of PARP1 at transcribed damaging sites. (a, b) U2OS-TRE cells transfected with TA-KR/tet-KR plasmids were light irradiated and allowed to recover for 0.5 h before fixation. Cells were stained with γH2AX antibody (a) or S9.6 antibody (b) (scale bar: 10 μm). γH2AX or R-loop foci frequency was quantified (n=3 independent experiments, 50 cells per replicate, Mean ± SD). (c) U2OS-TRE cells transfected with TA-KR with or without RNaseH plasmids were light irradiated and allowed to recover for 0.5 h before fixation. Cells were stained with S9.6 antibody (scale bar: 10 μm). R-loop foci frequency was quantified (n=3 independent experiments, 50 cells per replicate, Mean ± SD). Statistical analysis was done with the unpaired two-tailed student t-test. Source data are provided as a Source Data file.

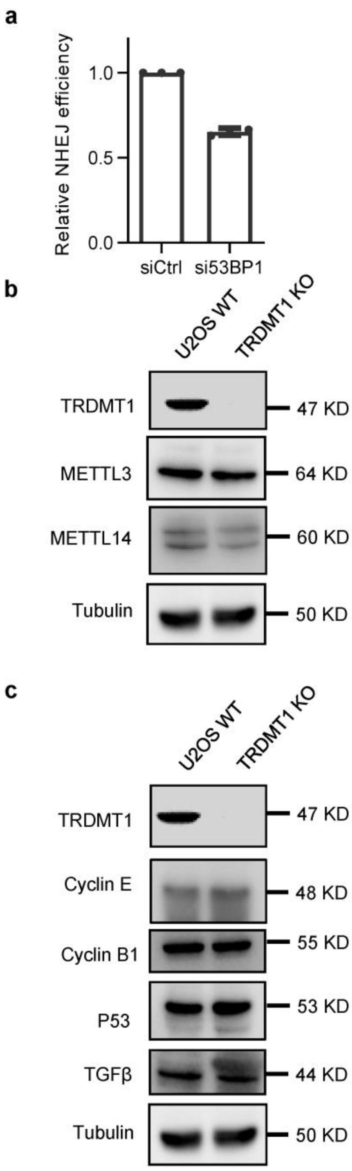

Supplementary Figure 5. The effect of TRDMT1 ko on the efficiency of alt-NHEJ is specifically dependent on the deficiency of RNA m5C by TRDMT1. (a) U2OS-TRE pretreated with 53BP1 siRNA or control siRNA were transfected with EJ5-GFP and I-SceI-Cherry plasmids. The fraction of GFP-positive cells in the Cherry-positive population was analyzed by flow cytometry (n = 3 independent experiments, mean ± SD). (b, c) WT and TRDMT1 KO U2OS-TRE cells were collected for WB. Indicated proteins were blotted with corresponding antibodies. Source data are provided as a Source Data file.

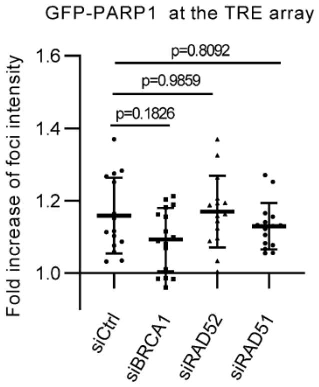

Supplementary Figure 6. PARP1 recruitment is not affected by RAD52 and RAD51. U2OS TRE cells pretreated with control siRNA or siBRCA1 or siRAD52 or siRAD51 were transfected with TA-KR and GFP-PARP1. The cells were light irradiated and allowed to recover for 0.5 h before fixation. Fold increase of GFP-PARP1 foci intensity was quantified. Mean intensity of PARP1 at TA-KR /mean intensity of background is shown (n=15 cells, Mean ±SEM). Statistical analysis was done with the one-way ANOVA. Source data are provided as a Source Data file.

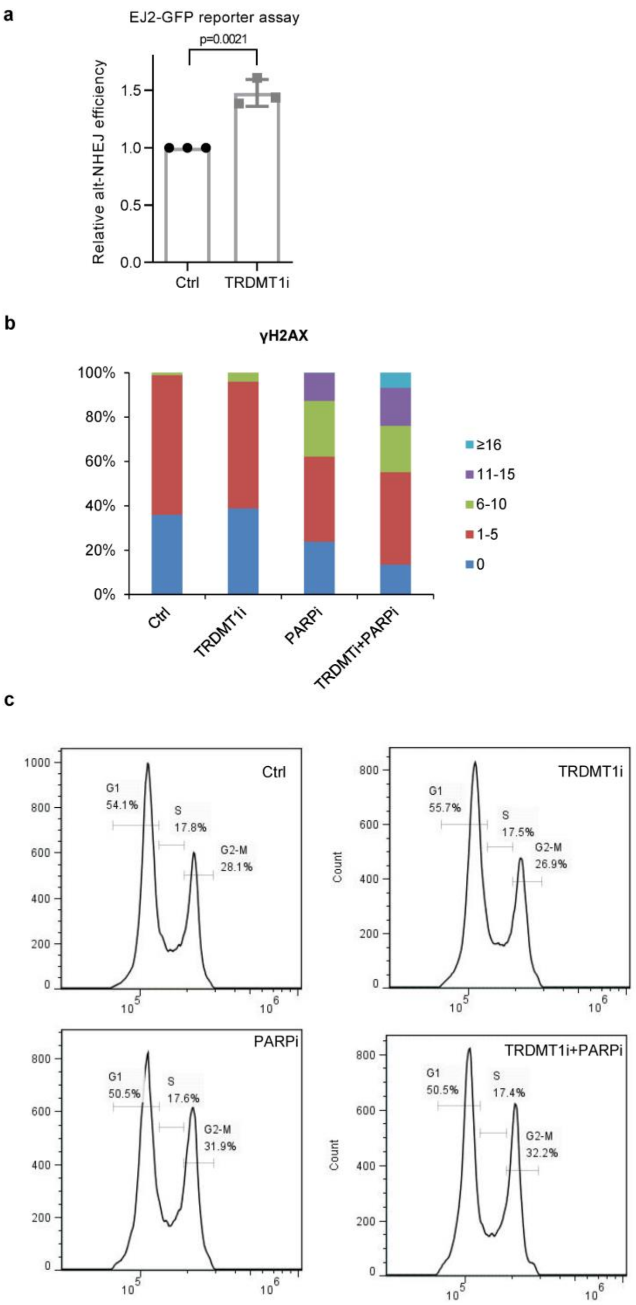

Supplementary Figure 7. Combination of TRDMT1i and PARPi leads to damage accumulation without affecting cell cycle progression. (a) U2OS-TRE cells were transfected with EJ2-GFP and I-SceI-Cherry plasmids. The cells were then treated with 2.5 μM TRDMT1i YW-1842. The fraction of GFP-positive cells in the Cherry-positive population was analyzed by flow cytometry (n = 3 independent experiments, mean ± SD). Statistical analysis was done with the unpaired two-tailed student t-test. (b and c) HS578T cells treated with 2.5 μM TRDMT1i YW-1842 or 1 μM PARPi olaparib were cultured for 1 day. The number of γ-H2AX foci per cell was quantified (b). Cells were stained with propidium iodide and analyzed by flow cytometry for cell cycle distribution (c). Source data are provided as a Source Data file.

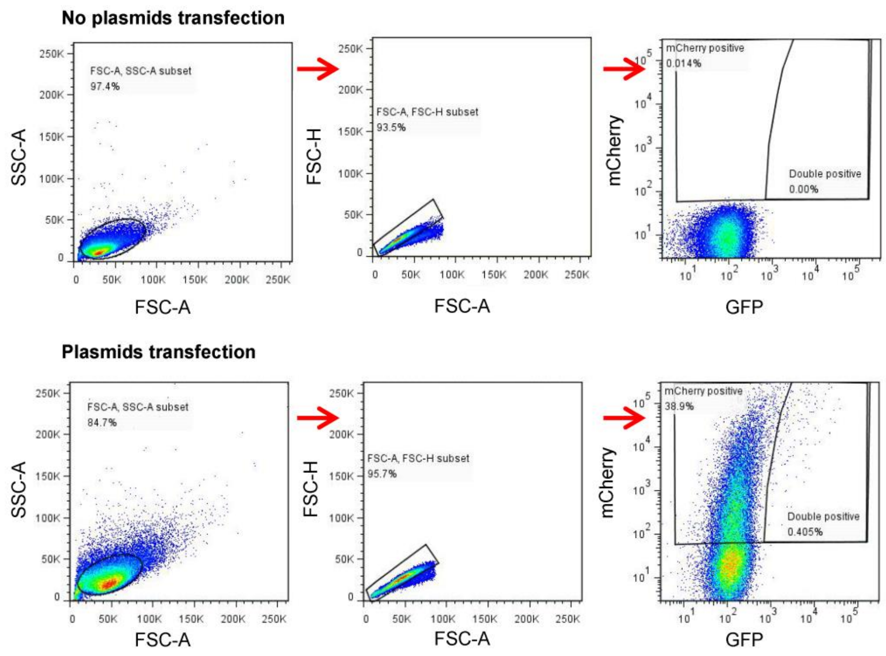

Supplementary Figure 8. Gating strategy used in flow cytometry analysis of EJ2-GFP Reporter Assay. U2OS WT cells with or without plasmids transfection were analyzed.

Supplementary Table 1. DNA repair proteins identified in the DNA:RNA hybrid pulldown fraction mass spectrometry analysis

		Average Intensity/Relative Intensity		
		Empty control	Unmodified hybrid	m5C modified hybrid
Unmodified hybrids preferential binding	PARP1	2.70E+08/1	3.70E+09/13.7	1.50E+09/5.6
	LIG3	3.20E+05/1	3.40E+07/106.3	1.20E+07/37.5
	XRCC1	1.60E+05/1	1.50E+07/93.8	3.90E+06/24.4
	XRCC5	8.00E+05/1	6.50E+06/8.1	2.70E+06/3.4
	XRCC6	1.50E+06/1	6.30E+06/4.2	3.20E+06/2.1
	DDX1	3.00E+04/1	5.90E+06/196.7	2.30E+06/76.7
	RPA1	0	3.90E+06/2.2	1.80E+06/1
	SUB1	6.40E+04/1	1.40E+06/21.9	9.00E+05/14.1
m5C modified hybrids preferential binding	HIST1H2BB	2.20E+07/1	2.30E+07/1	4.50E+07/2
	NAMPT	0	0	6.90E+04/1
	PKN2	0	0	5.80E+04/1
	RMI1	0	0	2.20E+04/1
Equal binding	RFC3	1.20E+05/1	6.80E+05/5.7	6.90E+05/5.8
	RPA3	0	9.70E+05/1	1.40E+06/1.4
	RPA2	0	6.10E+05/1.3	4.60E+05/1

Supplementary Table 2. **Oligos used in the EMSA assay and in vitro PARP1 activation assay**

Oligo name	Sequence
Oligo 1 (ssDNA 50 nts)	ATCATCACCATAACGTCGATGTATCAACTTCGATTAGTCACACC AATTAA
Oligo 2 (ssDNA 50 nts)	TTAATTGGTGTGACTAATCGAAGTTGATACATCGACGTTATGGT GATGAT
Oligo 3 (ssRNA 50 nts)	UUAAUUGGUGUGACUAAUCGAAGUUGAUACAUCGACGUUAU GGUGAUGAU
Oligo 4 (5mCRNA 50 nts)*	UUAAUUGGUGUGACUAAUCGAAGUUGAUACAUCGACGUUAU GGUGAUGAU

* Oligo 4 is methylated at each C

Supplementary Table 3. Abs used in the study.

Antibody	Species	Clone, Catalog no.	Company	Dilution
TRDMT1	Mouse monoclonal	D-9, sc-365001	Santa Cruz Biotechnology	1:100
m5C	Mouse monoclonal	33D3, ab10805	Abcam	1:200
S9.6	Mouse monoclonal	ENH001	Kerafast	1:200
GFP	Mouse monoclonal	11814460001	Roche	1:1000
PARP1	Mouse monoclonal	sc-56197, 5A5	Santa Cruz Biotechnology	1:500
PAR	Mouse monoclonal	MAB3192, 10H	EMD Millipore	1:1000
ɣH2AX, ser139	Mouse monoclonal	JBW301, 05–636	EMD Millipore	1:400
Polθ	Rabbit polyclonal	MBS9612322	Mybiosource	1:100
β-Actin	Mouse monoclonal	8H10D10	Cell Signaling Technology	1:5000
METTL3	Rabbit polyclonal	A8370	Abclonal	1:1000
METTL14	Rabbit polyclonal	A8530	Abclonal	1:500
TGFβ	Rabbit polyclonal	A2124	Abclonal	1:1000
P53	Mouse monoclonal	OP43, Ab-6	Oncogene	1:2000
Cyclin E	Mouse monoclonal	sc247, HE12	Santa Cruz Biotechnology	1:1000
Cyclin B	Rabbit polyclonal	sc594, H-20	Santa Cruz Biotechnology	1:1000
