# Supplementary material for: The RNA m5C modification in R-loops as an off switch of Alt-NHEJ
Source: Nat Commun. 2023 Sep 30;14:6114. doi: 10.1038/s41467-023-41790-w (PMC10542358; doi:10.1038/s41467-023-41790-w)
Supplement: Supplementary file 3 — Reporting Summary [file 41467_2023_41790_MOESM3_ESM.pdf]

nature portfolio

Corresponding author(s): Li Lan

Last updated by author(s): Sep 14, 2023

Reporting Summary

Nature Portfolio wishes to improve the reproducibility of the work that we publish. This form provides structure for consistency and transparency in reporting. For further information on Nature Portfolio policies, see our Editorial Policies and the Editorial Policy Checklist.

Statistics

For all statistical analyses, confirm that the following items are present in the figure legend, table legend, main text, or Methods section.

n/a	Confirmed	
☐	☒	The exact sample size (*n*) for each experimental group/condition, given as a discrete number and unit of measurement
☐	☒	A statement on whether measurements were taken from distinct samples or whether the same sample was measured repeatedly
☐	☒	The statistical test(s) used AND whether they are one- or two-sided<br>*Only common tests should be described solely by name; describe more complex techniques in the Methods section.*
☒	☐	A description of all covariates tested
☒	☐	A description of any assumptions or corrections, such as tests of normality and adjustment for multiple comparisons
☐	☒	A full description of the statistical parameters including central tendency (e.g. means) or other basic estimates (e.g. regression coefficient) AND variation (e.g. standard deviation) or associated estimates of uncertainty (e.g. confidence intervals)
☐	☒	For null hypothesis testing, the test statistic (e.g. *F*, *t*, *r*) with confidence intervals, effect sizes, degrees of freedom and *P* value noted<br>*Give P values as exact values whenever suitable.*
☒	☐	For Bayesian analysis, information on the choice of priors and Markov chain Monte Carlo settings
☒	☐	For hierarchical and complex designs, identification of the appropriate level for tests and full reporting of outcomes
☒	☐	Estimates of effect sizes (e.g. Cohen's *d*, Pearson's *r*), indicating how they were calculated

Our web collection on statistics for biologists contains articles on many of the points above.

Software and code

Policy information about availability of computer code

Data collection	FV1000 confocal software 4.2, Step one V2.3, ChemiDocTM MP imaging system
Data analysis	ImageJ 1.51k, Graphpad prism 6.01, Flowjo 10.6.2

For manuscripts utilizing custom algorithms or software that are central to the research but not yet described in published literature, software must be made available to editors and reviewers. We strongly encourage code deposition in a community repository (e.g. GitHub). See the Nature Portfolio guidelines for submitting code & software for further information.

Data

Policy information about availability of data

All manuscripts must include a data availability statement. This statement should provide the following information, where applicable:

- Accession codes, unique identifiers, or web links for publicly available datasets
- A description of any restrictions on data availability
- For clinical datasets or third party data, please ensure that the statement adheres to our policy

The bisulfite sequencing raw data has been deposited into NCBI's BioProject and are available with the accession number PRJNA833238 [https://www.ncbi.nlm.nih.gov/bioproject/PRJNA833238] and PRJNA984695 [https://www.ncbi.nlm.nih.gov/bioproject/?term=PRJNA984695]. The DRIP-seq raw data has been deposited into NCBI's BioProject with the accession number PRJNA833771 [https://www.ncbi.nlm.nih.gov/bioproject/?term=PRJNA833771]. The DNA:RNA hybrid pulldown fraction mass spectrometry raw data and search files have been deposited into the MassIVE data repository with accession code MSV000089337

[https://massive.ucsd.edu/ProteoSAFe/dataset.jsp?task=cc5a6de9723543cd978dfe9f1f6bcef5] and the PDX identifier is PXD045391. Source data are provided with this paper.

Human research participants

Policy information about studies involving human research participants and Sex and Gender in Research.

Reporting on sex and gender	N/A
Population characteristics	N/A
Recruitment	N/A
Ethics oversight	N/A

Note that full information on the approval of the study protocol must also be provided in the manuscript.

Field-specific reporting

Please select the one below that is the best fit for your research. If you are not sure, read the appropriate sections before making your selection.

☒ Life sciences ☐ Behavioural & social sciences ☐ Ecological, evolutionary & environmental sciences

For a reference copy of the document with all sections, see nature.com/documents/nr-reporting-summary-flat.pdf

Life sciences study design

All studies must disclose on these points even when the disclosure is negative.

Sample size	This study did not involve experiments with living animals. Thus, it was not necessary to define sample sizes in advance to ensure adequate statistical power. Sample size, number of replicates, error bars and statistical tests were chosen based on accepted practices in the field and stated in each figure legend. Generally, experiments were performed independently and reproduced using at least three biological replicates.
Data exclusions	No data was excluded in our analysis.
Replication	All experiments have been repeated 2-3 times as indicated in the figure legends.
Randomization	No animals or patients that would require randomization were involved. When individual cells were analyzed in cell populations, they were randomly selected from the populations.
Blinding	The investigators were not blinded during data collection. For western blots, FACS analysis, immunofluorescence, blinding is not possible because samples need to be loaded or analysed with knowledge of the sample identity.

Reporting for specific materials, systems and methods

We require information from authors about some types of materials, experimental systems and methods used in many studies. Here, indicate whether each material, system or method listed is relevant to your study. If you are not sure if a list item applies to your research, read the appropriate section before selecting a response.

Materials & experimental systems

n/a	Involved in the study	
☐	☒	Antibodies
☐	☒	Eukaryotic cell lines
☒	☐	Palaeontology and archaeology
☒	☐	Animals and other organisms
☒	☐	Clinical data
☒	☐	Dual use research of concern

Methods

n/a	Involved in the study	
☒	☐	ChIP-seq
☐	☒	Flow cytometry
☒	☐	MRI-based neuroimaging

Antibodies

Antibodies used	TRDMT1 Mouse monoclonal D-9, sc-365001 Santa Cruz Biotechnology 1:100<br>m5C Mouse monoclonal 33D3, ab10805 Abcam 1:200

S9.6 Mouse monoclonal ENH001 Kerafast 1:200
GFP Mouse monoclonal 11814460001 Roche 1:1000
PARP1 Mouse monoclonal sc-56197, 5A5 Santa Cruz Biotechnology 1:500
PAR Mouse monoclonal MAB3192, 10H EMD Millipore 1:1000
γH2AX, ser139 Mouse monoclonal JBW301, 05–636 EMD Millipore 1:400
Polθ Rabbit polyclonal MBS9612322 Mybiosource 1:100
β-Actin Mouse monoclonal 8H10D10 Cell Signaling Technology 1:5000
METTL3 Rabbit polyclonal A8370 Abclonal 1:1000
METTL14 Rabbit polyclonal A8530 Abclonal 1:500
TGFβ Rabbit polyclonal A2124 Abclonal 1:1000
P53 Mouse monoclonal OP43, Ab-6 Oncogene 1:2000
Cyclin E Mouse monoclonal sc247, HE12 Santa Cruz Biotechnology 1:1000
Cyclin B Rabbit polyclonal sc594, H-20 Santa Cruz Biotechnology 1:1000

Validation

All antibodies are commercially available and have been validated by suppliers and previous publications. Validation data is available in each of these company's website. Antibodies were used according to manufacture instructions.

Eukaryotic cell lines

Policy information about cell lines and Sex and Gender in Research

Cell line source(s)

U2OS TRE cells were generated from U2OS cells with integration of TRE repeats. Flp-in 293 cell line was purchased from Thermo Fisher (Cat#R75007). The HS578T and MDA-MB-231 cells are gifts from Dr. Leif W. Ellisen.

Authentication

Cell lines were not authenticated.

Mycoplasma contamination

No Mycoplasma contamination of cell lines. BM-cyclin (Sigma-Aldrich Cat 1079905001) was used during culture and removed before experiments.

Commonly misidentified lines
(See ICLAC register)

no commonly misidentified line were used

Flow Cytometry

Plots

Confirm that:

☒ The axis labels state the marker and fluorochrome used (e.g. CD4-FITC).

☒ The axis scales are clearly visible. Include numbers along axes only for bottom left plot of group (a 'group' is an analysis of identical markers).

☒ All plots are contour plots with outliers or pseudocolor plots.

☒ A numerical value for number of cells or percentage (with statistics) is provided.

Methodology

Sample preparation

U2OS cells were transfected with the EJ2-GFP and I-SceI-mCherry plasmids. Two days after transfection, the cells were collected for flow cytometry analysis

Instrument

LSR II

Software

flowjo 10.6.2

Cell population abundance

at least 10,000 cells were collected for each experiment

Gating strategy

The normal cell population was gated in P1 by SSC-A and FSC-A. The alt-NHEJ rate was then calculated as the ratio of GFP-positive cells number to mCherry –positive cells number.

☒ Tick this box to confirm that a figure exemplifying the gating strategy is provided in the Supplementary Information.
